# Ovsiankina’s Great Relief: How Supplemental Work during the Weekend May Contribute to Recovery in the Face of Unfinished Tasks

**DOI:** 10.3390/ijerph14121606

**Published:** 2017-12-20

**Authors:** Oliver Weigelt, Christine J. Syrek

**Affiliations:** 1 Work and Organizational Psychology, University of Hagen, 58084 Hagen, Germany; 2 Department of Organizational and Personnel Psychology, University of Rostock, D-18051 Rostock, Germany; 3 Work and Organizational Psychology, University of Trier, D-54286 Trier, Germany; syrek@uni-trier.de

**Keywords:** recovery, detachment, unfinished tasks, goal progress, relaxation, self-determination, autonomy need satisfaction, Ovsiankina effect, Zeigarnik effect, rumination

## Abstract

Unfinished tasks have been identified as a significant job stressor that impairs employee recovery after work. Classic experimental research by Ovsiankina has shown that people tend to resume yet unfinished tasks to satisfy their need for closure. We apply this notion to current working life and examine supplemental work after hours as a means to achieve peace of mind. We investigate how progress towards goal accomplishment through supplemental work may facilitate recovery in terms of psychological detachment, relaxation, autonomy, and mastery experiences. We conducted a week-level diary study among 83 employees over a period of 14 consecutive weeks, which yielded 575 observations in total and 214 matched observations of unfinished tasks, supplemental work during the weekend, progress, and recovery experiences. Unfinished tasks were assessed on Friday. Supplemental work and recovery experiences were assessed on Monday. Multilevel modeling analyses provide evidence that unfinished tasks at the end of the work week are associated with lower levels of detachment at the intraindividual level, tend to relate to lower relaxation, but are unrelated to autonomy and mastery. Progress towards finishing tasks during the weekend alleviates the detrimental effects of unfinished tasks on both kinds of recovery experiences. Supplemental work is negatively linked to detachment, but largely unrelated to the other recovery experiences.

## 1. Introduction

Recent research in the domain of occupational health psychology has noted that unfinished tasks at the end of the work week act as a threat to successful recovery during the weekend [1]. Theoretically, this line of current applied research is rooted in classic experimental laboratory research and the so-called Zeigarnik effect [2], which states that there is a memory advantage for unfinished tasks compared to finished tasks [3]. In the present study, we continue this line of current occupational stress research and apply another phenomenon rooted in classic experimental work on field theory [4]: The Ovsiankina effect [5]. Building on the Zeigarnik effect, Maria Ovsiankina found that participants in her experiments showed a strong tendency to resume tasks that had been interrupted and were therefore unfinished.

Given the ubiquity of opportunities to resume work in leisure time in today’s working life, for instance by using information and communication technology (ICT) [6,7], we suggest that the Ovsiankina effect may be highly relevant to understand supplemental work in leisure time and its effects on recovery. On the one hand, being preoccupied with work during leisure time is likely to impair recovery in terms of work to home interference [8] or lack of psychological detachment [9,10]. On the other hand, the presence of unachieved goals is also associated with lower levels of detachment during leisure time [11]—an empirical finding that is in line with the Zeigarnik effect. Employees are therefore likely to ruminate about work during the weekend when they have unfinished tasks [12], as the discrepancy between “wanting” and “having” [13] triggers perseverative thoughts about work in the absence of the necessity to do so [14]. Given that “the best way to terminate rumination is to attain the goal that is driving the rumination” ([15], p. 42), engaging in supplemental work after hours may be a means to achieve relief after finally finishing the unfinished tasks through supplemental work efforts during the weekend. In this sense, supplemental work after hours may be a double-edged sword. To further delve into the ambivalent role of supplemental work and to integrate these contradictory views, we draw on control theory [16] and examine the role of progress towards goal attainment as a moderator that explains when supplemental work after hours is beneficial rather than detrimental to recovery. In order to shed more light on the motives for supplemental work, we examine different reasons for work during the weekend and scrutinize their differential effects on recovery.

To gain a more comprehensive picture of how unfinished tasks and progress towards finishing them may affect recovery during the weekend, we focus on four major facets of recovery experiences that have been conceptualized in the literature [17], namely psychological detachment, relaxation, autonomy or control, and experiences of mastery. Whereas psychological detachment—defined as a ”sense of being away from the work situation” [9]—has been studied extensively in the literature for a review see [10], to date, the other facets of recovery experiences have attracted considerably less attention in prior research. However, empirical research has provided encouraging evidence that, in addition to detachment [18], mastery experiences [19], relaxation, and autonomy may be a mediating mechanism between job stressors and employee well-being. In this study, we therefore consider how unfinished tasks and progress towards finishing them through supplemental work during the weekend combine accumulate to affect all facets of recovery experiences. 

**Figure 1:**
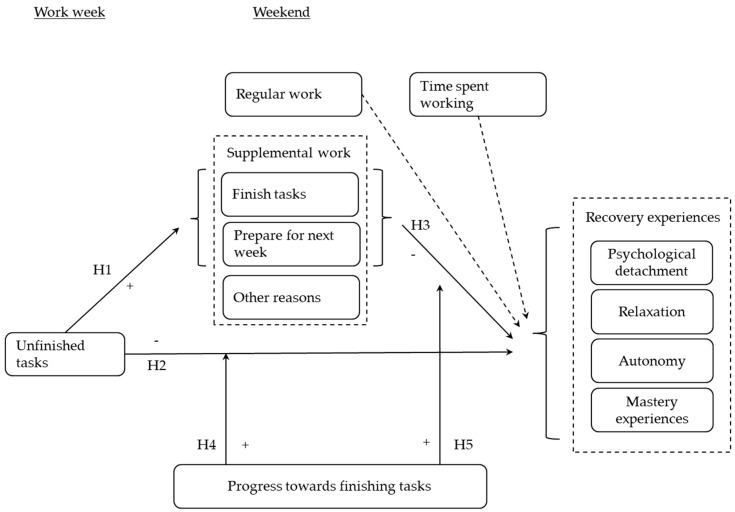
Theoretical model. H: Hypothesis; +: positive relationship; −: negative relationship.

Our study contributes to the literature in at least three ways. First, we examine how unfinished tasks relate to a broad range of recovery experiences, all of which are assumed to transmit detrimental effects from job stressors to employee well-being and health [17,20]. We further apply assumptions from basic research on rumination and the Ovsiankina effect to recovery from job stress in field settings. Second, we consider how supplemental work during the weekend relates to recovery experiences and examine whether supplemental work for different reasons yields differential effects on these facets of recovery. Third, we examine progress towards goal attainment—a concept derived from control theory (see also [15,16])—as a moderator that may reconcile opposite views on the role of supplemental work for recovery experiences, particularly for mentally switching off during the weekend. In essence, we integrate different theoretical perspectives, such as the Ovsiankina effect and elements of control theory to gain a more thorough understanding of the role of supplemental work after hours for employee recovery, and we examine boundary conditions of the detrimental effects of unfinished tasks on recovery experiences during the weekend. These insights may provide guidance on optimal ways of dealing with unfinished tasks at the end of the work week. Our focal theoretical model is depicted in Figure 1.

### 1.1. Linking Unfinished Tasks to Recovery

Researchers in the domain of occupational stress and recovery have recently revealed that unattained goals at the end of the work day [11] or unfinished tasks at the end of the work week [1,12] are associated with higher levels of perseverative thoughts about work during leisure time, in terms of either lower levels of psychological detachment and higher levels of affective rumination or problem-solving pondering. These applications of the Zeigarnik effect [2] to the domain of recovery from occupational stress suggest that not finishing tasks by the end of the work day or the work week comes at the cost of impaired recovery during leisure time. These psychological costs are incurred because employees are at least cognitively preoccupied with work [15] even in their leisure time and continue to call upon functional systems which have been strained during work [21]. In turn, rumination, that is, “conscious thoughts that revolve around a common instrumental theme and that recur in the absence of immediate environmental demands requiring [those] thoughts” ([15], p. 7), has been shown to be linked to several indicators of impaired well-being and health [22,23,24]. Hence, identifying ways to neutralize the detrimental effects of unfinished tasks on recovery seems to be a worthwhile endeavor, particularly from a practical perspective. Whereas recent research has provided evidence that interventions aimed at making plans for how to tackle unachieved goals before leaving work [11] may be one helpful strategy for highly involved employees, we examine yet another strategy of how to successfully cope with unfinished tasks: Resuming and actually finishing tasks after work by doing overtime through supplemental work. 

### 1.2. Supplemental Work after Hours: Applying the Ovsiankina Effect to Recovery from Occupational Stress during the Weekend

The empirical evidence cited above implies that facing unfinished tasks is aversive to the individual, and there is evidence for effects on affective well-being, too [25]. Unfinished tasks, therefore, call for doing something about it and resolve the tension associated with them in the service of closure, see [4]. In a series of classic experiments, Ovsiankina [5] interrupted her participants while they were working on a series of tasks and made them go on with the next task before finishing the current one. She found that participants tended to spontaneously resume working on the incomplete tasks as soon as they were given the slightest opportunity to do so—even if they were not allowed to and even if they initially did not like the task. From a theoretical perspective, resuming tasks offers the opportunity to finish or at least to make significant progress towards finishing uncompleted tasks and is an attractive option to eliminate the tension arising from unfinished tasks [5].

Whereas most participants in Ovsiankina’s experiments had to overcome considerable hurdles to continue working on unfinished tasks (e.g., waiting for the experimenter to apparently turn away), employees in today’s working life have plenty of opportunities to resume work tasks whenever and wherever they want to. Given the ubiquity of ICT and the fact that nowadays, in many professions and jobs, work is organized so that it can be carried out by means of telework [26], or technology-assisted supplemental work after hours (TASW) [7], while at home, the bar for resuming work after hours is set lower than ever [6]. We therefore examine whether the Ovsiankina effect can be applied to recovery from work stress in field settings. That is, whether unfinished tasks can account for resuming work after hours. Drawing on the Ovsiankina effect and the evidence cited above, we state:

**Hypothesis** **1.** *At the intraindividual level, unfinished tasks at the end of the work week will be positively associated with engagement in supplemental work during the weekend*.

We now turn to the recovery outcomes likely to be affected by unfinished tasks.

### 1.3. Linking Unfinished Tasks to the Full Range of Recovery Experiences

In the literature, it has been argued that recovery in terms of restoring or building up resources after work [21] may not depend on recovery activities per se, such as reading a book or going for a walk in the park, which apply equally to everyone. Instead, Sonnentag and Fritz [17] proposed that recovery activities unfold beneficial effects through enabling specific recovery experiences. In this sense, recovery experiences are assumed to be pivotal aspects of recovery that mediate the link between job stressors and employee well-being [17]. In line with this idea, Sonnentag and colleagues [18] found evidence for detachment as a linking mechanism between job stressors and well-being in terms of emotional exhaustion and a need for recovery in a multi-source survey study among Protestant pastors. In a similar way, in a cross-sectional survey study, Kinnunen et al. [19] found evidence of detachment, linking job demands and facets of employee fatigue. Although over the last decade a considerable volume of research on the role of detachment has accumulated [10], providing evidence that detachment plays a pivotal role in recovery, the other facets of the recovery experience questionnaire (relaxation, autonomy, and mastery experiences) have been largely neglected. However, theoretically, all of them have been conceptualized as psychological mechanisms linking job stressors to employee well-being and health [17,20]. Applying this general assumption to the effects of unfinished tasks, we expect that unfinished tasks at the end of the work week should be negatively related to all four facets of recovery experiences, namely detachment, relaxation, autonomy, and mastery experiences.

Given that psychological detachment is conceptualized in terms of the absence of thinking about work in leisure time [17] and that unfinished tasks have been positively linked to different facets of work-related rumination [1,12], expecting a negative link between unfinished tasks and detachment is straightforward. Moreover, this assumption is in line with the Zeigarnik effect and recent evidence on links between incomplete goals and detachment after work [11].

A similar logic can be applied to the recovery experience of relaxation. According to Sonnentag and Fritz ([17], p. 206), relaxation “is characterized by a state of low activation and increased positive affect”. Based on the logic of the Zeigarnik effect and empirical evidence linking unfinished tasks to affective rumination [12]—a state characterized by negatively laden thoughts about work [14,22], inner tension, and arousal—we expected that unfinished tasks are incompatible with the experience of relaxation during the weekend. 

Control over leisure time has been described as “a person’s ability to choose an action from two or more options” ([17], p. 207). In more general terms, such experiences have been referred to as satisfaction of the need for autonomy [20] as conceptualized in self-determination theory [27]. In terms of self-determination theory, autonomy (need satisfaction) refers to the “feeling that one’s activities are self-chosen and self-endorsed,” ([28], p. 326). Following the logic of the Ovsiankina effect, unfinished tasks are associated with an impulse to finish incomplete tasks. Experiencing an urge to resume work, in turn, undermines an employee’s free choice of spending time on leisure activities, which is at the heart of autonomy. Unfinished tasks are therefore likely to impair the experience of autonomy during leisure time. 

The experience of mastery has been described in terms of “opportunities for experiencing competence and proficiency” ([17], p. 206). Mastery experiences are challenges that do not overtax an individual’s capabilities. Being preoccupied with thinking about unfinished tasks and experiencing inner tension and negative arousal, however, is likely to consume substantial amounts of energy necessary to seek for and master other off-the-job challenges during leisure time. Therefore, unfinished tasks should be negatively related to mastery experiences during leisure time. 

Following the rationale of the Zeigarnik effect outlined above, we suggested that unfinished tasks at the end of the work week will trigger perseverative thoughts about work [1,11,12] and therefore interfere with all facets of recovery experiences. Given that detachment and relaxation are conceptually closely linked to (the absence of) rumination [14], we assume that associations are strongest for detachment and relaxation.

**Hypothesis** **2.** *At the intraindividual level, unfinished tasks at the end of the work week are negatively associated with (a) psychological detachment, (b) relaxation, (c) autonomy, and (d) mastery experiences during the weekend*.

### 1.4. Resuming Work during the Weekend: A Double-Edged Sword

We argued above that the presence of unfinished tasks is detrimental to recovery and employee well-being as it prevents mental disengagement [1,11,12]. Hence, taking action to finish unfinished tasks and resolve the tension associated with them appears to be warranted, given that finishing tasks allows employees to find peace of mind and hence to enjoy recovery activities. On the other hand, while people feel an urge to resume unfinished tasks, supplemental work after hours inevitably comes at the cost of spending time on work instead of time on recovery activities [29]. In this sense, supplemental work after hours is likely to be detrimental to recovery. Lending support to this assumption, Sonnentag [30] found that work-related activities during leisure time predicted impairment of well-being during the evening in a daily diary study. In another day-level diary study, Sonnentag and Zijlstra [29] found that work-related activities during off-job time predicted higher levels of need for recovery, and that need for recovery, in turn, predicted impaired well-being in terms of fatigue. 

However, scholars have also discussed the advantages to the individual arising from opportunities to continue work outside of regular business hours. New ways of working permit high degrees of temporal and spatial freedom including supplemental work after hours, for instance, working during the weekend from home. Empirically, opportunities for supplemental work outside of regular work hours per se do not necessarily impair well-being in terms of work–non-work balance, stress, and fatigue [31]. In a study on smartphone use and technology-assisted supplemental work (TASW), Derks and colleagues [32] found that employees issued by their employers with smartphones for professional use did not differ in terms of work–home interference (e.g., spillover of strain from work to home) from a group of non-users. Consequently, they ([32], p. 81) state: “Whether the impact of TASW on work–family balance is mainly positive or negative is still open for discussion”.

Given the inconsistent views and the ambivalent effects that engagement in supplemental work after hours may have with regard to recovery and employee well-being, we suggest considering the motives for supplemental work. From the perspective of self-determination theory [27,33], being compelled to do supplemental work to finish urgent tasks or prepare to meet external deadlines in the next week (cf. external or introjected regulation) may differ dramatically from resuming work for intrinsic reasons. Therefore, distinguishing between different motives for resuming work may explain why supplemental work per se may not always be detrimental to recovery. 

From the perspective of self-determination theory [33], supplemental work may differ in the degree to which it is self-imposed (e.g., intrinsic or integrated regulation) or initiated due to strong external demands (e.g., external or introjected regulation). Having to finish urgent tasks during the weekend or having to prepare for the next week may be typical motives for engaging in supplemental work, which would imply low levels of self-determination. In this sense, engaging in supplemental work is likely to be detrimental to recovery experiences. On the other hand, dealing with work-related issues in off-job time for intrinsic reasons may not yield the same effects that supplemental work for extrinsic reasons might. Therefore, we state here a formal hypothesis about supplemental work to prepare for the next week and to finish tasks. Moreover, to gain more insight, in our analyses, we also examine supplemental work for other reasons:

**Hypothesis** **3.** *At the intraindividual level, engaging in supplemental work particularly to finish tasks and to prepare for the next week during the weekend is negatively associated with (a) psychological detachment, (b) relaxation, (c) autonomy, and (d) mastery experiences during the weekend*.

Although the focus of our study is on *supplemental* work during the weekend, we also consider the role of *regular* work during the weekend as part of regular working hours. Considering regular work may offer theoretically and practically relevant insights, because regular work may be the least intrinsic type of work during the weekend from the perspective of self-determination theory. Our approach also offers the opportunity to compare the effects of supplemental work for different motives with the effects of regular work. Given the focus on supplemental work rather than regular work during the weekend, we do not state a formal hypothesis, but include and examine the effects of regular work vis-à-vis supplemental work. We also consider the amount of time spent working as a further predictor, which may be relevant. Engaging in supplemental or regular work likely comes at the expense of alternative leisure time activities in the service of recovery experiences. We therefore expect that the duration of either supplemental or regular work on average may be detrimental to all facets of recovery experiences. Both variables are included in Figure 1 using dotted lines to indicate that these links go beyond our focal hypotheses.

### 1.5. Considering the Role of Progress towards Goal Attainment

To further disentangle the factors involved in the detrimental and the beneficial effects of supplemental work on recovery, we draw on control theory [16] and introduce the concept of perceived progress towards goal attainment as a moderator variable, which carries the potential to reconcile inconsistent predictions regarding the effects of supplemental work after hours. Beyond moderating the link between supplemental work and recovery experiences, progress is also likely to neutralize the recovery impairing effects of unfinished tasks cited above.

One of the core assumptions of control theory is that individuals react negatively whenever there is a discrepancy between their standards or goals and actual outcomes [16,34]. While striving to resolve such discrepancies, monitoring progress is an aspect highly relevant to affective well-being, because progress conveys a sense of reducing the discrepancy between wanting and having—that is, approaching the goal. Applied to the phenomenon of recovery from work, unfinished tasks at the end of the work week are likely to be considered problematic by the individual employee because of the discrepancy and progress towards finishing unfinished tasks should reduce the (tension created by this) discrepancy [15]. Consequently, we propose that supplemental work after hours may not necessarily come at the cost of recovery, but may even *facilitate* recovery, once individuals have succeeded in finishing tasks or at least having made significant progress towards goal attainment [15]. With regard to unfinished tasks, we expect that the negative link between unfinished tasks will be neutralized if significant progress is made during the weekend. With regard to the link between supplemental work and recovery, we expect progress to also act as a moderator. Combining the rationale of the Ovsiankina effect and aspects of control theory [16,34], above, we have argued that progress towards finishing unfinished tasks alleviates the detrimental effects of unfinished tasks on recovery experiences. In other words, high levels of progress towards goal attainment imply elimination of unfinished tasks during the weekend. Hence, the detrimental effects on recovery experiences should be substantially reduced once significant progress has been made. Unless employees are preoccupied with thinking about unfinished tasks, they can cherish recovery experiences without restrictions.

**Hypothesis** **4.** *Progress towards finishing tasks moderates the link between unfinished tasks at the end of the work week and (a) detachment, (b) relaxation, (c) autonomy, and (d) mastery experiences during the weekend. The links between unfinished tasks and recovery experiences will be strongest for low levels of progress towards finishing tasks*.

With regard to supplemental work, we follow a similar line of reasoning. We assume that, although spending time on supplemental work is incompatible with focal recovery experiences, it may cease to constrain recovery or even facilitate detachment, relaxation, autonomy, and mastery experiences once employees have made significant progress. In line with the assumption that cognitive activation of unfinished tasks is decreased when individuals resume progress towards the task [15], supplemental work may reduce inner tension if employees make significant progress. Similarly, Syrek et al. [12] argue that goal progress reduces uncertainty, which frees resources to subsequently engage in recovery activities. Extending prior research on supplemental work and in line with control theory, we expect that progress towards finishing tasks alleviates the recovery-impairing effects outlined above.

**Hypothesis** **5.** *At the intraindividual level, progress towards finishing tasks during the weekend moderates the link between supplemental work and (a) psychological detachment, (b) relaxation, (c) autonomy, and (d) mastery experiences during the weekend. The links between supplemental work and recovery experiences will be strongest for low levels of progress towards finishing tasks*.

## 2. Materials and Methods

### 2.1. Procedure

Most experience sampling research has studied recovery either during holidays or in the evenings during the work week [35]. Research on recovery during the weekend has only just started to accumulate. Given that the weekend comprises a period of more than a couple of hours of rest from work it is one major opportunity for a wide range of recovery experiences, but also for opportunities to reflect on work and engage in supplemental work during leisure time. To complement prior research on day-level recovery experiences, we conducted a week-level diary study over a period of 14 consecutive weeks. We chose this long period as opportunities for and occurrence of actual engagement in supplemental work during leisure time may not vary very much within a time frame a couple of days or of two or three weekends. After completing a general survey capturing demographics and potential control variables, participants received invitations to participate in brief online surveys every Friday afternoon after leaving work and every Monday before starting work over a period of three months.

### 2.2. Sample

The participants in our sample were employees enrolled on a psychology program at a German university that offers distance-learning courses. Our initial dataset consisted of 575 matched observations from 83 participants. This figure is equivalent to 50% out of 1162 possible complete observations. On average, each participant provided seven matched Friday–Monday weekly diary surveys. This sample was used to examine the Ovsiankina effect stated in Hypothesis 1 and the effects of supplemental work. The full sample consisted of 72% women and 28% men. Two persons did not indicate their gender. The average age in the focal sample was 36.94 years (SD = 9.60), ranging from 21 to 65. One-third had children. Most participants had either general qualifications for university entrance (75%) or advanced technical college entrance qualifications (16%). Our participants worked in different organizations. They came from diverse industries, mainly from healthcare (24%), the service sector (24%), education (15%), the public security sector (7%), public administration (7%), the manufacturing industry (4%), commerce (9%), and other industries (10%). On average, participants worked 32 hours per week (SD = 10.4). Seventy-four had a permanent employment contract. The majority of participants did not have a managerial position (69%). Given that either regular or supplemental work during the weekend was a prerequisite for our focal analyses on progress in unfinished tasks during the weekend (it cannot be assessed meaningfully unless participants have engaged in work) and that not all participants engaged in regular or supplemental work during the weekend during all the weekends studied, we confined our analyses to predicting recovery experiences to a subset of the initial sample. We included all observations for weekends when either regular or supplemental work during the weekend was present. The focal sample consisted of complete weekly reports on all focal week-level variables of this study by 65 employees; this yielded 215 matched weekly observations eligible for our focal analyses (37% of the initial sample). See Table 1 for details on sample sizes for each wave of data collection.

### 2.3. Measures 

Unless otherwise stated, our scales ranged from 1 (totally disagree) to 5 (totally agree). Unfinished tasks were reported on Friday, and supplemental work and recovery experiences during the weekend were assessed on Monday. 

#### 2.3.1. Unfinished Tasks

Unfinished tasks at the end of the week were measured on Friday afternoon using a five-item scale developed and validated by Syrek et al. [12]. A sample item is “I have not finished important tasks that I had planned to do this week”. 

#### 2.3.2. Supplemental Work during the Weekend

In the Monday surveys, participants reported whether they had engaged in supplemental work during the weekend. We applied the following item: “During the weekend, have you worked on job-related tasks?” Participants responded using the following response options, which referred to their motives for working during the weekend, if applicable. The answer options were as follows: “No,” “Yes, in order to finish unfinished tasks from the last work week,” “Yes, in order to prepare for the forthcoming work week,” and “Yes, for other reasons.” To differentiate supplemental work during leisure time from regular work as part of shiftwork, there was a fifth checkbox option: “Yes, I was on duty during the weekend or I have worked as part of my regular working hours.” Participants could check any option or any combination of reasons for working during the weekend. However, most observations referred to only a single type of work during the weekend. Frequencies were as follows: 53 observations of supplemental work to prepare for the next week, 21 observations of supplemental work to finish tasks, 94 observations of supplemental work for other reasons, and 60 observations of regular work during the weekend. We created four dummy variables for each motive (1 = checked, 0 = not checked) and included all of them as indicators of supplemental work in the focal analyses. To use supplemental work as a criterion variable examining the Ovsiankina effect, we summed up the items referring to supplemental work to (1) finish unfinished tasks and (2) prepare for the next week (bivariate correlation *r* = 0.21, *p* < 0.001) to form a composite score that depicts the degree of engagement in supplemental work for extrinsic reasons. We combined the two items to create a non-binary outcome, which allows for differentiating higher from lower degrees of supplemental work. Conceptually, it may not always be easy to differentiate between catching up and preparing for the next week. Plausibly, finishing tasks from the current week may often be in order to prepare for the next week, when results are due. The moderate but substantial correlation of the two items is consistent with our rationale. We therefore assume that our composite score reflects the degree of engaging in supplemental work in a meaningful way and depicts a higher degree of variance than analyzing single binary items. The composite supplemental work score ranged from 0 (no supplemental work at all) to 2 (supplemental work for both reasons). A score of 1 was equivalent to one type of supplemental work. We further captured the amount of time spent on work in hours to use it as a control variable besides unfinished tasks at the end of the work week. This item was meant to capture the duration or intensity of either supplemental or regular work.

#### 2.3.3. Progress towards Finishing Tasks

If participants had engaged in work during the weekend, they rated the extent to which they had made progress towards finishing their unfinished tasks. The item was “If you have worked on work-related tasks during this weekend, how much progress have you made?” The item was rated on a scale ranging from 0 (“I have made no progress at all”) to 4 (“I have finished all of the unfinished tasks”).

#### 2.3.4. Recovery Experiences during the Weekend

We measured recovery using the detachment, relaxation, and mastery experiences subscales of the recovery experience questionnaire [17] adapted to the purposes of our study. Each facet was measured by four items. Respective sample items were “During this weekend I forgot about work,” “During this weekend I used [my] time to relax,” and “During this weekend I sought out intellectual challenges.” In line with our more general approach to autonomy, we applied three items adapted from Sheldon et al. [28] and van den Broeck et al. [36] to capture autonomy need satisfaction. The three items were “During this weekend I had the feeling that my choices were based on my true interests and values,” “During this weekend I had the feeling that my choices expressed my ‘true self,’” and “During this weekend I had the feeling that I can be myself.” To make sure that a parsimonious and yet reliable measure of autonomy during the weekend was applied, four of the items of the scale by Sheldon et al. [28] and five items of the scale by van den Broeck et al. [36] were included in the baseline survey to factor-analyze the comprehensive set of nine items from both original instruments. The items quoted above turned out to be the highest-loading items that captured the autonomy factor most reliably. The set of items capturing autonomy in the weekend surveys was then restricted to three items for reasons of parsimony. Means, standard deviations, and correlation matrices of all measures at the intraindividual level are presented in Table 2. Descriptive information and correlations at the interindividual level are presented in Table 3.

### 2.4. Analytic Strategy

Given the nested structure of our data, we applied multilevel modeling for repeated measures [37]. Effects on Level 1 refer to fluctuations within persons over multiple weeks. Within the context of our study, intraclass correlations (ICCs) for our focal variables depict fluctuations within individuals across time, which is a prerequisite for multilevel modeling. The ICCs (1) for recovery experiences ranged from 0.26 to 0.48 and provide evidence that two-thirds to three-quarters of the variance is within persons. Additional analyses yielded ICCs (1) of 0.55 for unfinished tasks, 0.30 hours spent working, and 0.53 for progress (see Table 2 for all ICCs). Our multilevel approach is therefore warranted [38].

In line with the recommendations for centering predictors in experience sampling studies [39], unfinished tasks were centered at the person-mean. Our analyses consequently refer to deviations from the average level of each variable over multiple weeks for each person [39]. For progress towards finishing tasks, we deviated from this approach and did not center, but kept the original metric, as the value of zero for our measure was meaningful (no progress at all) and as we were interested in absolute rather than relative differences between different weekends within persons. Hence, we did not study whether making more progress than the previous weekend was associated with a difference with regard to recovery experiences, but we analyzed whether making considerable progress compared to little or no progress during the current weekend predicted higher levels of recovery experiences. We applied the same logic to the motives for supplemental work after hours, regular work, and time spent working as further week-level predictors. We expected that characteristic average levels [35] or “chronic” levels of recovery experiences likely differ between employees. We further wanted to take into account that the links between unfinished tasks and progress with recovery experiences might be interindividually different, too. Therefore, we specified random intercepts and random slopes models. Random slopes for the focal predictors is also straightforward when studying interactions at the intraindividual level [40].

We applied the “nlme” library [41] for the R statistics package and followed recommendations for specifying random coefficient models using R [42]. Models were built step by step beginning with the least complex null models. As a precaution against potential violations of the multilevel modeling assumptions, we specified autocorrelation and heteroscedasticity (see [42] in preliminary analyses). We further included linear time trends (i.e., growth trajectories in recovery experiences over time). If these specifications improved the fit of the statistical models, they were retained in the model. Otherwise, they were omitted for reasons of parsimony. Generally, adding autocorrelation, heteroscedasticity, or growth slopes did not change the patterns of results for our focal predictors.

Given that we aimed to study the role of regular work and given that a substantial portion of the focal sample captured occasions of regular work during the weekend, we had to disentangle progress through supplemental work and progress through regular work. Although progress may yield the same effects on recovery experiences irrespective of whether it was achieved through supplemental or regular work, our hypotheses refer to supplemental work only. The same rationale applies to time spent working during the weekend, which may either refer to supplemental work or regular work. To examine Hypotheses 4 and 5, we therefore included two-way interactions of the type of work (supplemental work to prepare, to finish tasks, or for other reasons as well as regular work) and a three-way interaction of unfinished tasks, progress, and type of work. A significant three-way interaction indicates that unfinished tasks and progress through supplemental work yield effects that are different from progress through regular work.

## 3. Results

### 3.1. Examining the Ovsiankina-Effect

To examine whether the Ovsiankina effect can be applied to unfinished tasks and supplemental work during the weekend, we ran multilevel regression analyses and regressed our supplemental work score (based on supplemental work to prepare and to finish tasks) on unfinished tasks at the end of the work week. The results of the multilevel regression analysis are presented in Table 4. At the intraindividual level, unfinished tasks are positively related to engagement in supplemental work (γ = 0.04, *p* < 0.03). In line with Hypothesis 1, when intraindividually higher levels of unfinished tasks were present on Friday, employees had a stronger inclination to engage in supplemental work to catch up or to prepare for next week. Adding the person-mean of unfinished tasks as a covariate at the interindividual level, as a measure of chronic levels of unfinished tasks, did not predict interindividual differences in engagement in supplemental work.

### 3.2. Linking Unfinished Tasks and Supplemental Work to Recovery Experiences

Using the full sample (*n* = 575), in a first step, we examined intraindividual links between unfinished tasks on Friday and recovery experiences during the weekend. In line with Hypothesis 2a, we found a negative link at the intraindividual level between unfinished tasks and detachment, when unfinished tasks were entered as the only predictor (γ = −0.11, *p* = 0.03). In the next step, we included the motives for engaging in work as dummy coded predictors besides unfinished tasks. Our final models for the four facets of recovery experiences are depicted in Table 5. We did not find evidence that detachment, relaxation, autonomy, and mastery were intraindividually related to (higher levels of) unfinished tasks at the end of the work week, when engaging in supplemental work is taken into account (|γ| < 0.07, *p* > 0.10). We further studied the effects of unfinished tasks using the focal sample of those observations when participants worked during the weekend (*n* = 215). The results for the focal sample are presented in Table 6. With regard to Hypothesis 2, we found unfinished tasks to be negatively related to detachment (γ = −0.46, *p* = 0.03). Unfinished tasks also tended to be related to relaxation (γ = −0.42, *p* = 0.05), but did not yield associations with autonomy and mastery experiences. Our analyses therefore provide only partial support for Hypothesis 2a regarding links to detachment and no support for Hypotheses 2b–d regarding links to the other recovery experiences.

Examining Hypothesis 3 on the links between supplemental work during the weekend and recovery experiences, we analyzed the full sample and entered regular work and the motives for engaging in supplemental work as predictors besides unfinished tasks. The results are presented in Table 5. With regard to supplemental work during the weekend, finishing tasks for different motives, preparing for the work week ahead and other reasons were uniformly negatively related to detachment (all γ < −0.81, *p* < 0.001). Supplemental work to prepare for the next week tended to be negatively related to relaxation (γ = −0.28, *p* = 0.07). Conversely, supplemental work for other reasons was even positively related to mastery experiences (γ =−0.39, *p* = 0.02), a finding that suggests that some kinds of supplemental work after hours might even be beneficial for recovery experiences, rather than detrimental. Contrary to Hypothesis 3c, supplemental work did not yield associations with autonomy. Engagement in regular work was negatively related to detachment, relaxation, and autonomy, but unrelated to mastery experiences. 

### 3.3. Progress towards Finishing Tasks as a Moderator

**Figure 2:**
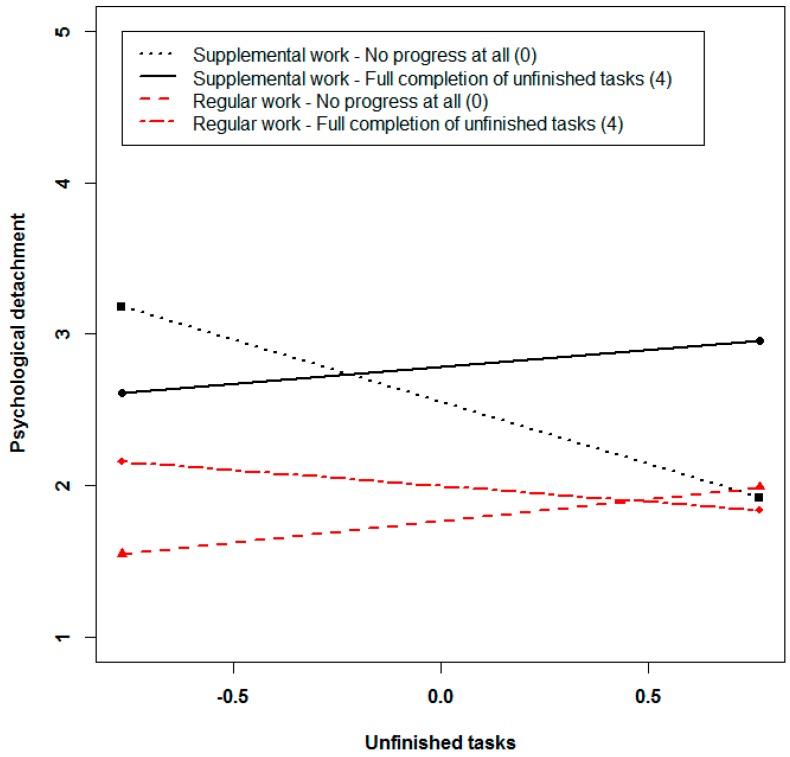
Three-way interactive effects of unfinished tasks, type of work, and progress during the weekend predicting psychological detachment.

To examine Hypothesis 4, we analyzed the focal sample of observations when participants engaged in any type of work during the weekend (*n* = 215). Besides the main effects of unfinished tasks and progress towards finishing them, we entered a series of interactions of each focal predictor with the type of work (supplemental work to prepare, supplemental work to finish tasks, supplemental work for other reasons, and regular work) to estimate the effects for each type of supplemental work in a non-confounded way. We included the interaction of unfinished tasks and progress towards finishing tasks to examine whether progress buffers the detrimental effects of unfinished tasks on recovery experiences. To make sure that our results are interpretable in terms of progress through supplemental (rather than regular) work, we included three-way interactions of type of work with the focal unfinished tasks and progress interaction. Results are presented in Table 6. In line with Hypotheses 4a and 4b, we found evidence for moderation for detachment (γ = 0.24, *p* < 0.01) and relaxation (γ = 0.22, *p* = 0.02). Furthermore, we found a three-way interaction of unfinished tasks, progress, and regular work (γ = −0.43, *p* < 0.01) predicting detachment, which suggests that the moderating effects of progress differ between occasions of regular work vs. all other types of work (supplemental work). The pattern of the interaction is illustrated in Figure 2.

In the case of supplemental work, the association between unfinished tasks and detachment is strongest when employees make no progress at all. On the other hand, decline in detachment is not dependent upon higher levels of unfinished tasks, when an employee achieves full completion of unfinished tasks at the same time. Interestingly, detachment is highest for a combination of intraindividually low levels of unfinished tasks and low levels of progress. In the case of regular work, unfinished tasks are not related to detachment, irrespective of progress towards finishing tasks. In sum, the pattern of results regarding Hypothesis 4a suggests that progress through supplemental work buffers the detrimental effects of unfinished tasks on detachment.

**Figure 3:**
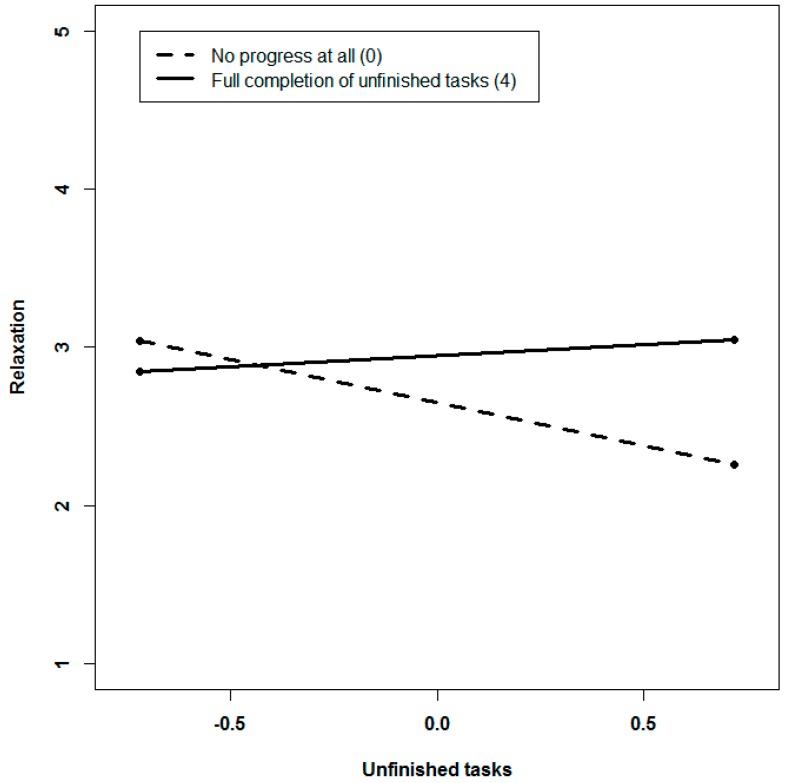
Interactive effects of unfinished tasks and progress during the weekend predicting relaxation.

With regard to relaxation, we found no evidence for three-way interactions. The two-way interaction regarding Hypothesis 4b is described in Figure 3. Whereas higher levels of unfinished tasks are associated with lower levels of relaxation in the face of no progress, relaxation is not affected by the level of unfinished tasks when full completion of unfinished tasks has been achieved during the weekend. When intraindividually low levels of unfinished tasks are present, progress towards finishing tasks does not make a difference with regard to relaxation—a pattern of results that does not differ by the type of work through which progress is achieved. Analysis of simple slopes provides evidence that the negative link between unfinished tasks drops to nonsignificant when employees make substantial progress towards finishing tasks during the weekend.

**Figure 4:**
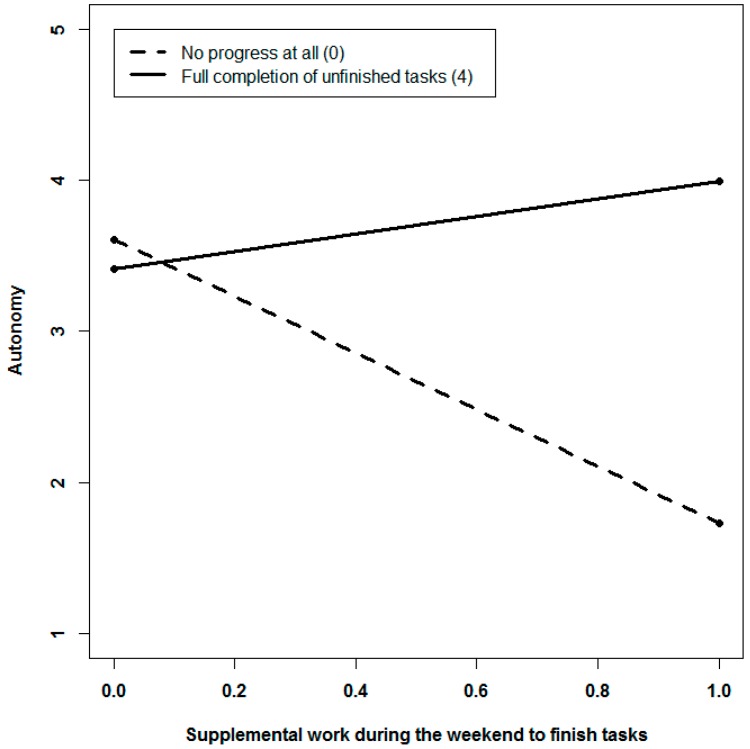
Interactive effects of supplemental work to finish tasks (yes/no) and progress during the weekend predicting autonomy.

We followed the same approach to examine whether the effects of engaging in supplemental work on recovery experiences were buffered by high levels of progress as suggested in Hypothesis 5. In line with Hypothesis 5c, we found that progress alleviates the negative link between supplemental work to finish tasks and autonomy (γ = 0.67, *p* = 0.02) only. The pattern of the interaction is shown in Figure 4. If employees engage in supplemental work to finish tasks, they experience less autonomy during the weekend. This negative association holds only when employees make no progress during the weekend. However, if they make significant progress, supplemental work to finish tasks is unrelated to experiencing autonomy. Predicting the other facets of recovery experiences, supplemental work for any reason and progress did not interact (We reran our focal models controlling for demographics. Given that our analyses focused on associations at the intrainidividual level and given that the results were equivalent to those of our focal models, we omitted demographics for reasons of parsimony).

## 4. Discussion

In this study, we set out to examine how unfinished tasks and progress towards finishing them through supplemental work during the weekend contribute to recovery experiences in terms of psychological detachment from work, relaxation, autonomy, and mastery experiences. We scrutinized our hypotheses derived from an integration of the Ovsiankina effect and control theory based on a comprehensive week-level longitudinal study.

### 4.1. Theoretical Implications

First, our study provides evidence that the Ovsiankina effect is one mechanism that accounts for why employees engage in supplemental work during the weekend. More specifically, unfinished tasks are positively associated with resuming work after hours, a finding which is consistent with the empirical evidence from the classic experimental studies [5]. In this sense, our results suggest that, in working life in the 21st century, the implications of the Zeigarnik effect and the Ovsiankina effect may be more topical than ever. Hence, understanding the contingencies of these mechanisms is key to successfully managing work–life balance from the perspectives of employers and employees. At a minimum, unfinished tasks can be considered one major driver of recovery impairment besides other phenomena implied by the ubiquity of ICT in employees’ private lives (e.g., receiving cues from colleagues, expectations of permanent availability) [6,43]. Our results also inform the emerging literature on ICT use and how it relates to recovery and health, e.g., [44].

Second, drawing on control theory [16,34], we examined boundary conditions for the detrimental effects of unfinished tasks on recovery experiences during the weekend. Consistent with the Zeigarnik effect, unfinished tasks have been shown to be associated with a lack of switching off and with rumination after work [1,11,12]. In this study, we extended the scope beyond rumination and a lack of detachment to the recovery experiences of relaxation, autonomy, and mastery. We studied how these states are affected by unfinished tasks at the week-level over a period of several months. Our broad approach is in line with recent conceptualizations of how leisure is linked to well-being [20]. In sum, we found that unfinished tasks are associated with lower levels of recovery, particularly in terms of detachment. However, relaxation tended to be only negatively related to unfinished tasks, while autonomy and mastery were not affected by unfinished tasks. In line with our assumptions derived from control theory [16,34], making significant progress towards finishing tasks by engaging in supplemental work during the weekend neutralized these recovery-impairing effects. As illustrated in Figure 2 and Figure 3, the recovery inhibiting effects of unfinished tasks on detachment and relaxation are neutralized when employees make significant progress towards finishing tasks or even complete their unfinished tasks during the weekend. Making progress seems to imply a reduction in uncertainty, and feelings of confidence that the task has been adequately finished emerge [13], freeing resources that can be used to detach from work and relax during the remaining leisure time. Consequently, our metaphor of experiencing relief after having made significant progress seems to be quite an accurate description of the dynamics at work. Our results suggests that behaviors that result in attaining incomplete goals might contribute to recovery by eliminating the cause of perseverative thoughts [15]. Although they require some effort in the short run, they may turn out to be adaptive and beneficial after a while.

Third, we examined whether engaging in supplemental work during the weekend for different reasons, such as finishing tasks from the previous week or preparing for the next week, makes a difference. We further concurrently studied the effects of different types of supplemental work and regular work. Although in the full sample all reasons for supplemental work negatively predicted detachment, a differential pattern emerged for the other recovery experiences: Engaging in supplemental work—no matter what the reasons were—did not significantly impair relaxation, autonomy, or mastery. Our results therefore suggest that the detrimental effects of engaging in supplemental work are largely confined to impairing switching off during the weekend. In contrast, engaging in regular work was consistently linked to lower levels of detachment, relaxation, and autonomy—a finding that highlights that our distinction between working for reasons that range from largely extrinsic (regular work) to intrinsic regulation (supplemental work for other reasons) may be relevant for understanding when and why working during the weekend comes at the cost of recovery [33]. Interestingly, working for reasons other than catching up or needing to prepare for the next week even yielded beneficial effects for mastery experiences. This result suggests that working, for instance, for intrinsic reasons [33] out of interest in job-related tasks may be conducive to positive recovery experiences [45] and contribute to an experience of competence need satisfaction, but this comes at the cost of not switching off. This finding corroborates empirical evidence on the potentially beneficial effects of problem-solving pondering as a positive way of being preoccupied with work-related issues during leisure time [12,46]. In this sense, our results indicate that the value of challenging activities during leisure time for recovery and health [47], besides activities fostering hedonic well-being in terms of relaxation and detachment, cannot be underestimated. Experiencing “a fair day’s work” during leisure time, for instance through progress by means of supplemental work, may convey a sense of meaning and therefore be a powerful driver of eudaimonic well-being [20,48,49].

Fourth, we have questioned that supplemental work after hours is unambiguously detrimental to employee recovery. Although, as shown in Figure 4, we found that supplemental work to finish tasks was associated with lower levels of autonomy, our results suggest that these behaviors may not yield adverse effects, as long as they result in significant progress towards goal accomplishment. One can speculate whether significant progress enabled employees to perceive their remaining leisure time as more under their control—as being freed from the urge to finish tasks. The feeling of relief that accompanies the perception of progress [12,13] might trigger the perception of having more control over the subsequent time off work. In this sense, our study corroborates findings from prior research that new ways of working and opportunities for supplemental work per se may not necessarily result in impaired recovery from job stressors [31] and may not contribute to a further build-up of strain. Although supplemental work at first sight comes at the cost of not disengaging from work, it does not necessarily hamper recovery experiences, particularly when it results in progress.

### 4.2. Practical Implications

Given the ubiquity of unfinished tasks and the abundance of cues reminding individuals about the things left undone during their leisure time (e.g., by ICT) [6], we need to identify leverage points for interventions from a practical perspective. Smit [11] recently introduced an intervention aimed at improving coping with incomplete goals by means of making plans for which steps to take next to complete tasks. He provides empirical evidence that at least highly involved individuals benefited from this strategy. Besides changing perceptions of unfinished tasks before they spill over into leisure time, the results of our study imply that tools which facilitate *effectively* finishing work in leisure time may be another approach worthy of consideration, as this strategy may minimize the potentially detrimental effects of hours worked during leisure time and at the same time foster progress towards attaining goals. Therefore, scheduling a clearly defined time window for finishing a specific task through supplemental work after hours may be a viable alternative to bear the tension emanating from unfinished tasks while not at work. In this sense, *successful* supplemental work provides a basis for detachment and relaxation thereafter.

Finally, performance expectations of supervisors have been shown to moderate the positive link between unfinished tasks and rumination [1]. Hence, setting realistic performance expectations may be a viable option to avoid detrimental effects on recovery experiences before they even arise.

### 4.3. Strengths and Limitations

Although this study features some considerable strengths in terms of study design and analysis (week-level diary study over a period of four months, separation of predictors and criteria, applying reliable measures, and focusing on effects at the intraindividual level), we must also concede some limitations.

First, our data come from a single source and are confined to self-reports: However, the focal interactions cannot easily be attributed to third variables. Although retrospective reports on the recovery experiences over a period of two days may not be as accurate as momentary assessments [50], within our analyses at the intraindividual level, these biases may be constant within persons and hopefully not affect results dramatically. 

Second, although we have examined the hypotheses on the Ovsiankina effect and the role of supplemental work based on the full sample, with regard to the focal sample, we have a high percentage of missing data, particularly due to the fact that not every participant engaged in either supplemental or regular work after hours during all weekends studied. Our analyses regarding Hypotheses 4 and 5, however, relied on engaging in work during the weekend as a prerequisite to make progress. Still, on average the analyses refer to nearly four observations per participant, providing for enough power to examine random effects and find evidence for interactions at the intraindividual level. To examine whether the focal sample might differ in terms of individual differences, we regressed membership in the focal (vs. the full) sample on demographics, but did not find individual differences in terms of demographics to be related to membership in the focal sample—a finding that does not strongly indicate self-selection. Furthermore, comparing the full sample and the focal sample suggests that bivariate correlations and the pattern of results regarding Hypothesis 2 yielded quite consistent results. In this sense, the focal sample may provide a fairly accurate and representative picture of the full sample during the period studied. However, we have studied a sample of highly educated individuals, who have taken the effort to participate in our study over the course of four months. Therefore, our results may not necessarily generalize to samples that differ in terms of education or involvement.

Third, although most of our scales were highly reliable at the interindividual and the intraindividual levels, our measure of autonomy reached only acceptable to good reliability and may therefore capture heterogeneous aspects within one scale. Although our one-item measure of progress towards finishing tasks appears to be parsimonious and face-valid, it does not allow rigorously probing reliability. The pattern of correlations with the other variables, however, suggests that we measured something clearly distinct from unfinished tasks, supplemental work after hours, and recovery experiences. However, future research should aim at more reliable measurement, using a multi-item scale to capture progress.

Fourth, although we captured focal predictors and criteria at different points in time and our analyses basically refer to the intraindividual level, we cannot establish a causal effect from unfinished tasks on Friday to recovery experiences during the weekend. Future research might aim to corroborate our findings using even more rigorous designs and analytical approaches outlined in the future research section below.

Despite these shortcomings, to us this study appears to be a well-balanced compromise between the need for scientific rigor and aspects of feasibility in applied field research. From our perspective, taking all limitations into account, the study still offers a non-trivial step forward to understanding the dynamics of recovery as implied by the Zeigarnik and the Ovsiankina effects. 

### 4.4. Avenues for Future Research

In this study, we have taken a broad perspective on four aspects of recovery experiences proposed by Fritz and Sonnentag [51]. However, more recent conceptualizations of how leisure experiences contribute to subjective well-being [20] propose additional aspects which may be relevant, but go beyond the four-facet approach developed by Sonnentag and Fritz [17]. More specifically, in their DRAMMA model Newman and colleagues [20] propose that meaning [48] and satisfaction of the need for affiliation [27] may be relevant aspects to consider besides detachment, relaxation, autonomy, and mastery experiences. For instance, as evident in the unexpected positive link between supplemental work for other reasons and mastery experiences, working after hours, although straining, may convey a sense of meaning to the employee. Future research could follow up and further elaborate on the distinction between supplemental work for intrinsic vs. extrinsic motives, which might also integrate other intrinsically motivated forms of work in off-job time like volunteering [47,52,53].

Throughout this manuscript, we have argued that unfinished tasks are associated with inner tension and rumination [2,12,13]. Our results on the buffering role of progress imply that inner tension will drop once unfinished tasks have been finished or at least significant progress has been made during the weekend. Future research might examine the dynamics implied by our line of reasoning more rigorously using experience-sampling data, monitoring progress, tension, and rumination several times a day throughout the weekend. Discontinuous growth curve modeling [54] techniques offer opportunities for even better accounting for the complexities and dynamics involved during the weekend.

Although our analyses do not provide strong evidence that individuals tending to resume work in off-job time differ from those who do not in terms of demographics, future research might consider individual differences. For instance, Smit [11] found that highly involved individuals [55] benefited most from an intervention targeted at coping with unattained goals. Further research has also considered the buffering effects of self-control [56] for detaching from job stressors. In this sense, a worthwhile avenue for future research might integrate self-control and self-regulation perspectives with our rationale derived from control theory to gain further insights into why and when employees engage in supplemental work.

We have stressed in our study the role of the individual reasons to resume work during the weekend. However, future research might also study supplemental work behaviors during the weekend embedded in the context of organizational practices and norms to engage in supplemental work [44,57]. One might also consider the role of job design (e.g., unrealistic workload) in fostering supplemental work and the factors that explain whether employees prefer supplemental work to behaviors aimed at challenging suboptimal job design [58] in terms of proactive work behavior [59] or job crafting [60].

## 5. Conclusions

Since the classic experiments of Ovsiankina on the resumption of interrupted tasks in the 1920s, the nature of work has changed dramatically. In light of new ways of working and the high accessibility of information and communication technology to most employees in industrialized countries, we face an abundance of opportunities for resuming unfinished job-related tasks during leisure time—even, or particularly, during the weekend. Applying the human tendency to resume unfinished tasks—the Ovsiankina effect—to the domain of recovery from job stress, we examined the role of progress towards goal attainment as a variable that might buffer the detrimental effects of unfinished tasks on facets of recovery. In line with control theory, our results based on a week-level diary study provide evidence that progress neutralizes the detrimental effects on the recovery experiences of psychological detachment and relaxation. Given the intriguing finding that supplemental work after hours may actually facilitate relief from the burden of unfinished tasks and hence may also have a bright side, we look forward to resuming this line of research in the future.

## Figures and Tables

**Table 1:** Number of matched observations per week for each of the 14 waves.

Wave Number	1	2	3	4	5	6	7	8	9	10	11	12	13	14
Full sample (*n* = 575)	42	46	47	47	33	45	49	45	43	49	44	45	35	5
Focal sample (*n* = 215)	23	20	13	15	15	17	19	12	14	18	17	22	7	3

**Table 2:** Means, standard deviations, and correlations between study variables at the intraindividual level.

Variable	M	SD	ICC	*α_Level 1_*	*α_Level 2_*	1	2	3	4	5	6	7	8	9	10	11
1. Unfinished tasks	2.40	1.22	0.55	0.93	0.99		0.08	**0.19**	**0.15**	**−0.15**	0.04	**−0.28**	**−0.14**	−0.11	−0.13	**−0.15**
2. Hours spent working	2.58	5.53	0.30			−0.03		−0.11	−0.01	**0.59**	0.02	**0.23**	**−0.41**	**−0.29**	−0.06	**−0.21**
3. SW to prepare ^a^						**0.11**	0.08		0.10	**−0.33**	**−0.18**	−0.05	−0.08	0.02	−0.02	−0.01
4. SW to finish tasks ^a^						**0.09**	0.08	**0.21**		**−0.20**	−0.11	**0.14**	−0.10	−0.04	−0.10	0.06
5. SW for other reasons ^a^						**−0.13**	**0.69**	**−0.12**	−0.08		**−0.29**	**0.27**	**−0.26**	**−0.19**	−0.09	**−0.24**
6. Regular work ^a^						0.03	**0.13**	−0.02	−0.02	−0.11		0.08	−0.06	0.03	**0.15**	0.08
7. Progress ^b^	2.64	1.42	0.53			**−0.28**	**0.23**	−0.05	**0.14**	**0.27**	0.08		−0.11	0.08	−0.01	0.07
8. Detachment	3.36	1.25	0.48	0.91	0.99	−0.06	**−0.46**	**−0.20**	**−0.16**	**−0.41**	**−0.19**	**−0.11**		**0.65**	**0.34**	**0.42**
9. Relaxation	3.05	1.08	0.26	0.92	0.95	−0.03	**−0.19**	−0.04	−0.06	**−0.17**	−0.03	0.08	**0.47**		**0.24**	**0.51**
10. Autonomy	2.80	1.05	0.48	0.83	0.97	−0.07	−0.02	0.01	−0.05	−0.02	**0.11**	−0.01	**0.23**	0.08		**0.37**
11. Mastery experiences	3.64	1.03	0.32	0.85	0.96	**−0.13**	**−0.15**	−0.04	0.01	**−0.18**	0.02	0.07	**0.38**	**0.42**	**0.27**	

Correlations below the diagonal are week-level correlations for the full sample (*n* = 575), and correlations above the diagonal are week-level correlations for the focal sample (*n* = 215). M = mean; Correlations in bold face are significant at *p* < 0.05; SD = standard deviation; ICC = intra-class correlation; *α_Level 1_* = Multilevel alpha at the intraindividual level; *α_Level 2_* = Multilevel alpha at the interindividual level; SW = supplemental work; ^a^ 0 male; 1 female; ^b^ *n* = 215.

**Table 3:** Means, standard deviations, and correlations between study variables at the interindividual level.

Variable	M	SD	1	2	3	4	5	6	7	8	9	10	11	12	13	14	15
1. Gender ^a^	0.72	0.45		−0.14	−0.12	−0.03	0.02	0.14	−0.15	0.15	−0.10	0.16	−0.16	−0.21	−0.09	−0.06	0.08
2. Age in years	36.94	9.60	−0.16		**0.51**	**0.35**	−0.02	−0.08	0.12	**0.38**	0.20	−0.22	0.07	−0.07	−0.11	0.00	0.17
3. Tenure in years	6.00	5.94	−0.06	**0.48**		0.20	−0.04	0.17	0.00	**0.39**	−0.05	0.00	−0.01	−0.11	−0.18	0.07	0.21
4. Parental status ^b^	0.33	0.47	−0.02	**0.34**	**0.26**		0.14	0.00	0.17	0.10	0.02	−0.14	−0.11	−0.05	**−0.25**	0.04	−0.04
5. Unfinished tasks ^c^			0.06	0.04	0.11	0.19		−0.02	0.14	0.04	0.17	−0.14	**−0.28**	−0.17	−0.18	−0.24	−0.19
6. Hours spent working ^c^			0.08	−0.06	0.09	0.04	−0.05		−0.06	0.10	0.16	**0.72**	0.13	**−0.53**	−0.24	**−0.25**	0.04
7. SW to prepare ^c^			−0.13	0.08	−0.03	0.18	0.10	0.05		0.13	0.05	**−0.25**	−0.01	−0.17	−0.14	−0.09	0.02
8. SW to finish tasks ^c^			0.13	0.33	**0.32**	0.10	0.03	0.14	0.16		−0.13	−0.08	0.17	−0.19	−0.16	0.03	0.00
9. SW for other reasons ^a^			−0.09	0.15	−0.07	0.05	0.13	**0.23**	0.12	−0.08		−0.23	0.07	−0.18	0.00	−0.09	0.12
10. Regular work ^c^			0.09	−0.17	−0.05	−0.10	−0.15	**0.74**	−0.16	−0.04	−0.15		0.18	**−0.36**	−0.07	−0.12	0.11
11. Progress ^c^			−0.16	0.07	−0.01	−0.11	**−0.28**	0.13	−0.01	0.17	0.07	0.18		0.01	0.22	0.18	0.17
12. Detachment ^c^			−0.18	−0.01	−0.04	−0.06	−0.13	**−0.58**	**−0.25**	**−0.22**	**−0.26**	**−0.41**	0.01		**0.70**	**0.58**	**0.29**
13. Relaxation ^c^			−0.03	−0.20	**−0.25**	−0.21	−0.15	**−0.24**	−0.14	−0.14	−0.03	−0.10	**0.22**	**0.57**		**0.56**	**0.47**
14. Autonomy ^c^			−0.09	0.02	0.02	0.03	−0.17	−0.16	−0.04	0.05	−0.04	−0.06	0.18	**0.37**	**0.31**		**0.43**
15. Mastery experiences ^c^			0.04	0.15	0.10	−0.03	−0.13	0.02	0.02	0.00	0.10	0.08	0.17	**0.22**	**0.45**	**0.39**	

Correlations below the diagonal are week-level correlations for the full sample (*n* = 83, *n* = 81 for demographics), and correlations above the diagonal are week-level correlations for the focal sample (*n* = 65, *n* = 63 for demographics). Correlations in bold type are significant at *p* < 0.05. *SD* = standard deviation. SW = supplemental work. ^a^ 0 male, 1 female. ^b^ 0 no children, 1 one or more children. ^c^ Person-mean over time.

**Table 4:** Results from multilevel analysis predicting supplemental work during the weekend in the full sample (*n* = 575).

Parameter	Supplemental Work			
	Estimate	SE	*t*	
Intercept	0.14	0.03	4.88	
Interindividual level				
Person-mean unfinished tasks	0.05	0.03	1.65	
Intraindividual level				
Unfinished tasks (UT)	0.04	0.02	2.23	*
Variance components				
Level 2 intercept variance	0.23			
Unfinished tasks slope variance	0.07			
Level 1 intercept variance	0.30			
Deviance (*df*)	381.21			(7)
AIC	395.21			
BIC	425.69			

SE = standard error. *df* = degrees of freedom. * *p* < 0.05. Deviance = (−2 Residual Log Likelihood).

**Table 5:** Results from multilevel analysis predicting recovery experiences during the weekend in the full sample (*n* = 575).

Parameter	Detachment				Relaxation				Autonomy				Mastery			
	Estimate	*SE*	*t*		Estimate	*SE*	*t*		Estimate	*SE*	*t*		Estimate	*SE*	*t*	
Intercept	3.72	0.09	42.72		3.22	0.08	39.72		3.67	0.09	40.45		2.84	0.08	33.89	
Unfinished tasks (UT)	−0.07	0.05	−1.24		0.05	0.06	0.84		−0.03	0.05	−0.64		−0.05	0.05	−1.04	
SW—Preparing next week (SW prep) ^a^	−0.81	0.14	−5.88	***	−0.28	0.15	−1.83	†	−0.16	0.13	−1.21		0.08	0.15	0.53	
SW—Finishing tasks (SW finish) ^a^	−0.82	0.24	−3.48	***	−0.36	0.26	−1.40		−0.07	0.22	−0.33		−0.24	0.25	−0.97	
SW—Other reasons (SW other) ^a^	−0.95	0.15	−6.37	***	−0.24	0.17	−1.42		−0.03	0.14	−0.22		0.39	0.16	2.44	*
Regular work ^a^	−1.45	0.12	−11.65	***	−0.72	0.14	−5.23	***	−0.43	0.12	−3.61	***	−0.15	0.13	−1.15	
Variance components																
Level 2 intercept variance	0.66				0.56				0.71				0.60			
Unfinished tasks slope variance	0.20				0.20				0.15				0.01			
Level 1 intercept variance	0.78				0.89				0.73				0.86			
Deviance (*df*)	1498.78		(10)		1571.47		(10)		1440.31		(10)		1615.25		(10)	
AIC	1518.78				1591.47				1460.31				1635.25			
BIC	1562.32				1635.02				1503.86				1678.80			

*SE* = standard error. *df* = degrees of freedom. † *p* < 0.10. * *p* < 0.05. *** *p* < 0.001. Deviance = (−2 Residual Log Likelihood). Analyses refer to the full sample (*n* = 575). ^a^ dummy-coded variables: 0 = no. 1 = yes.

**Table 6:** Results from multilevel analysis predicting recovery experiences during the weekend in the focal sample (*n* = 215).

Parameter	Detachment				Relaxation				Autonomy				Mastery			
	Estimate	*SE*	*t*		Estimate	*SE*	*t*		Estimate	*SE*	*t*		Estimate	*SE*	*t*	
Intercept	3.02	0.27	11.37		2.66	0.27	10.00		4.01	0.26	15.57		3.08	0.30	10.25	
Unfinished tasks (UT)	−0.46	0.21	−2.23	*	−0.42	0.21	−1.96	†	−0.12	0.18	−0.64		0.17	0.21	0.83	
SW—Preparing next week (SW prep) ^a^	−0.47	0.46	−1.01		0.43	0.48	0.90		−0.39	0.42	−0.93		−0.15	0.51	−0.30	
SW—Finishing tasks (SW finish) ^a^	−1.68	1.04	−1.61		−0.87	1.13	−0.77		−2.01	0.92	−2.18	*	−1.51	1.10	−1.37	
SW—Other reasons (SW other) ^a^	−1.03	0.54	−1.89	†	0.55	0.55	0.99		−0.02	0.52	−0.03		−0.27	0.60	−0.44	
Regular work ^a^	−1.58	0.82	−1.93	†	−0.39	0.83	−0.47		−0.76	0.77	−0.98		−0.77	0.90	−0.85	
Time worked during the weekend in hours	0.07	0.07	0.93		0.10	0.08	1.23		−0.03	0.07	−0.47		0.01	0.08	0.17	
Progress towards finishing tasks	0.05	0.10	0.50		0.17	0.10	1.72	†	−0.19	0.09	−2.21	*	−0.13	0.10	−1.35	
UT × progress	0.24	0.09	2.67	*	0.22	0.09	2.43	*	0.05	0.08	0.65		−0.07	0.09	−0.72	
Time worked × progress	−0.03	0.01	−1.83	†	−0.01	0.01	−0.68		0.01	0.01	0.83		−0.01	0.02	−0.64	
Time worked × SW prep	0.00	0.06	0.04		−0.05	0.07	−0.68		0.05	0.06	0.84		−0.04	0.07	−0.54	
Time worked × SW finish	−0.03	0.07	−0.38		−0.07	0.07	−0.98		−0.02	0.06	−0.28		0.04	0.07	0.56	
Time worked × SW other	−0.07	0.07	−0.93		−0.08	0.08	−1.08		−0.03	0.07	−0.53		0.04	0.08	0.53	
Time worked × regular work	−0.05	0.07	−0.71		−0.12	0.07	−1.68	†	0.00	0.06	0.02		0.01	0.07	0.16	
Progress × SW prep	0.03	0.15	0.19		−0.21	0.15	−1.36		0.08	0.13	0.63		0.21	0.16	1.33	
Progress × SW finish	0.40	0.31	1.29		0.24	0.33	0.73		0.67	0.28	2.42	*	0.44	0.32	1.41	
Progress × SW other	0.30	0.18	1.65		−0.20	0.19	−1.07		0.12	0.17	0.74		0.20	0.19	1.01	
Progress × regular work	0.33	0.24	1.36		0.08	0.25	0.34		0.18	0.22	0.81		0.28	0.26	1.09	
UT × SW prep	−0.82	0.53	−1.55		−0.99	0.54	−1.82	†	0.20	0.46	0.43		−0.40	0.57	−0.70	
UT × SW finish	0.58	1.23	0.47		−0.32	1.31	−0.24		0.92	1.09	0.85		−2.24	1.36	−1.64	
UT × SW other	0.95	1.03	0.92		0.13	1.07	0.12		1.42	0.93	1.52		−0.38	1.06	−0.36	
UT × regular work	1.10	0.42	2.64	**	0.59	0.40	1.47		−0.06	0.34	−0.18		−0.07	0.40	−0.18	
UT × progress × SW prep	0.12	0.20	0.62		0.29	0.20	1.43		−0.14	0.17	−0.84		0.06	0.21	0.30	
UT × progress × SW finish	−0.26	0.37	−0.72		−0.01	0.39	−0.02		−0.34	0.33	−1.04		0.67	0.41	1.66	
UT × progress × SW other	−0.42	0.43	−0.99		−0.19	0.44	−0.43		−0.66	0.38	−1.76	†	−0.07	0.42	−0.15	
UT × progress × regular work	−0.43	0.14	−2.99	**	−0.21	0.14	−1.52		−0.02	0.12	−0.19		0.04	0.14	0.25	
Variance components	0.84				0.77				0.90				1.00			
Level 2 intercept variance	0.39				0.28				0.17				0.13			
Progress slope variance	0.35				0.31				0.24				0.23			
Level 1 intercept variance	0.60				0.73				0.58				0.71			
Deviance (*df*)	533.76		(17)		555.30		(17)		502.79		(17)		552.73		(17)	
AIC	567.76				589.30				538.79				586.73			
BIC	624.98				646.52				599.37				643.95			

*SE* = standard error. *df* = degrees of freedom. † *p* < 0.10. * *p* < 0.05. ** *p* < 0.01. Deviance = (−2 Residual Log Likelihood). Analyses refer to the focal sample (*n* = 215). ^a^ dummy-coded variables: 0 = no. 1 = yes.

## References

[1] Syrek C.J., Antoni C.H. (2014). Unfinished tasks foster rumination and impair sleeping—Particularly if leaders have high performance expectations. J. Occup. Health Psychol..

[2] Zeigarnik B., Ellis W.D. (1938). On finished and unfinished tasks. A Source Book of Gestalt Psychology.

[3] Zeigarnik B. (1927). Das Behalten erledigter und unerledigter Handlungen [Remembering finished and unfinished tasks]. Psychol. Forsch..

[4] Lewin K., Cartwright D. (1951). Field Theory in Social Science: Selected Theoretical Papers.

[5] Ovsiankina M. (1928). Untersuchungen zur Handlungs-und Affektpsychologie. Psychol. Forsch..

[6] Ďuranová L., Ohly S. (2016). Persistent Work-Related Technology Use, Recovery and Well-Being Processes: Focus on Supplemental Work after Hours.

[7] Fenner G.H., Renn R.W. (2004). Technology-Assisted Supplemental Work: Construct Definition and a Research Framework. Hum. Resour. Manag..

[8] Van Hooff M.L.M., Geurts S.A.E., Kompier M.A.J., Taris T.W. (2006). Work-home interference: How does it manifest itself from day to day?. Work Stress.

[9] Etzion D., Eden D., Lapidot Y. (1998). Relief from job stressors and burnout: Reserve service as a respite. J. Appl. Psychol..

[10] Sonnentag S., Fritz C. (2015). Recovery from job stress: The stressor-detachment model as an integrative framework. J. Organ. Behav..

[11] Smit B.W. (2015). Successfully leaving work at work: The self-regulatory underpinnings of psychological detachment. J. Occup. Organ. Psychol..

[12] Syrek C.J., Weigelt O., Peifer C., Antoni C.H. (2017). Zeigarnik’s sleepless nights: How unfinished tasks at the end of the week impair employee sleep on the weekend through rumination. J. Occup. Health Psychol..

[13] Martin L.L., Tesser A., McIntosh W.D., Wegner D.M., Pennebaker J.W., Wegner D.M., Pennebaker J.W. (1993). Wanting but not having: The effects of unattained goals on thoughts and feelings. Handbook of Mental Control.

[14] Cropley M., Zijlstra F.R.H., Langan-Fox J., Cooper C.L. (2011). Work and rumination. Handbook of Stress in the Occupations.

[15] Martin L.L., Tesser A., Wyer R.S.J. (1996). Some ruminative thoughts. Ruminative Thoughts.

[16] Carver C.S., Scheier M.F. (1982). Control theory: A useful conceptual framework for personality-social, clinical, and health psychology. Psychol. Bull..

[17] Sonnentag S., Fritz C. (2007). The recovery experience questionnaire: Development and validation of a measure for assessing recuperation and unwinding from work. J. Occup. Health Psychol..

[18] Sonnentag S., Kuttler I., Fritz C. (2010). Job stressors, emotional exhaustion, and need for recovery: A multi-source study on the benefits of psychological detachment. J. Vocat. Behav..

[19] Kinnunen U., Feldt T., Siltaloppi M., Sonnentag S. (2011). Job demands–resources model in the context of recovery: Testing recovery experiences as mediators. Eur. J. Work Organ. Psychol..

[20] Newman D.B., Tay L., Diener E. (2014). Leisure and subjective well-being: A model of psychological mechanisms as mediating factors. J. Happiness Stud..

[21] Meijman T.F., Mulder G., Drenth P.J.D., Thierry H., de Wolff C.J., Drenth P.J.D., Thierry H., de Wolff C.J. (1998). Psychological aspects of workload. Handbook of Work and Organizational: Work Psychology.

[22] Cropley M., Plans D., Morelli D., Sütterlin S., Inceoglu I., Thomas G., Chu C. (2017). The association between work-related rumination and heart rate variability: A field study. Front. Hum. Neurosci..

[23] Cropley M., Zijlstra F.R.H., Querstret D., Beck S. (2016). Is work-related rumination associated with deficits in executive functioning?. Front. Psychol..

[24] Querstret D., Cropley M. (2012). Exploring the relationship between work-related rumination, sleep quality, and work-related fatigue. J. Occup. Health Psychol..

[25] Gabriel A.S., Diefendorff J.M., Erickson R.J. (2011). The relations of daily task accomplishment satisfaction with changes in affect: A multilevel study in nurses. J. Appl. Psychol..

[26] Allen T.D., Golden T.D., Shockley K.M. (2015). How effective is telecommuting? Assessing the status of our scientific findings. Psychol. Sci. Public Interest.

[27] Deci E.L., Ryan R.M. (2000). The “what” and “why” of goal pursuits: Human needs and the self-determination of behavior. Psychol. Inq..

[28] Sheldon K.M., Elliot A.J., Kim Y., Kasser T. (2001). What is satisfying about satisfying events? Testing 10 candidate psychological needs. J. Pers. Soc. Psychol..

[29] Sonnentag S., Zijlstra F.R.H. (2006). Job characteristics and off-job activities as predictors of need for recovery, well-being, and fatigue. J. Appl. Psychol..

[30] Sonnentag S. (2001). Work, recovery activities, and individual well-being: A diary study. J. Occup. Health Psychol..

[31] Nijp H.H., Beckers D.G.J., van de Voorde K., Geurts S.A.E., Kompier M.A.J. (2016). Effects of new ways of working on work hours and work location, health and job-related outcomes. Chronobiol. Int..

[32] Derks D., ten Brummelhuis L.L., Zecic D., Bakker A.B. (2014). Switching on and off…: Does smartphone use obstruct the possibility to engage in recovery activities?. Eur. J. Work Organ. Psychol..

[33] Gagné M., Deci E.L. (2005). Self-determination theory and work motivation. J. Organ. Behav..

[34] Carver C.S., Scheier M.F. (1990). Origins and functions of positive and negative affect: A control-process view. Psychol. Rev..

[35] Ilies R., Aw S.S.Y., Pluut H. (2015). Intraindividual models of employee well-being: What have we learned and where do we go from here?. Eur. J. Work Organ. Psychol..

[36] Van den Broeck A., Vansteenkiste M., De Witte H., Soenens B., Lens W. (2010). Capturing autonomy, competence, and relatedness at work: Construction and initial validation of the Work-Related Basic Need Satisfaction scale. J. Occup. Organ. Psychol..

[37] Raudenbush S.W., Bryk A.S. (2002). Hierarchical Linear Models: Applications and Data Analysis Methods.

[38] Singer J.D., Willett J.B. (2003). Applied Longitudinal Data Analysis: Modeling Change and Event Occurrence.

[39] Enders C.K., Tofighi D. (2007). Centering predictor variables in cross-sectional multilevel models: A new look at an old issue. Psychol. Methods.

[40] Bauer D.J., Preacher K.J., Gil K.M. (2006). Conceptualizing and testing random indirect effects and moderated mediation in multilevel models: New procedures and recommendations. Psychol. Methods.

[41] Pinheiro J.C., Bates D.M. (2000). Mixed-Effects Models in S and S-PLUS.

[42] Bliese P.D., Ployhart R.E. (2002). Growth modeling using random coefficient models: Model building, testing, and illustrations. Organ. Res. Methods.

[43] Dettmers J., Bamberg E., Seffzek K. (2016). Characteristics of extended availability for work: The role of demands and resources. Int. J. Stress Manag..

[44] Park Y., Fritz C., Jex S.M. (2011). Relationships between work-home segmentation and psychological detachment from work: The role of communication technology use at home. J. Occup. Health Psychol..

[45] Martin L.L., Tesser A., Sanna L.J., Chang E.C., Sanna L.J., Chang E.C. (2006). Extending the goal progress theory of rumination: Goal reevaluation and growth. Judgments Over Time: The Interplay of Thoughts, Feelings, and Behaviors.

[46] Vahle-Hinz T., Mauno S., de Bloom J., Kinnunen U. (2017). Rumination for innovation? Analysing the longitudinal effects of work-related rumination on creativity at work and off-job recovery. Work Stress.

[47] Mojza E.J., Sonnentag S., Bornemann C. (2011). Volunteer work as a valuable leisure-time activity: A day-level study on volunteer work, non-work experiences, and well-being at work. J. Occup. Organ. Psychol..

[48] Ryff C.D. (1989). Happiness is everything, or is it? Explorations on the meaning of psychological well-being. J. Pers. Soc. Psychol..

[49] White M.P., Dolan P. (2009). Accounting for the Richness of Daily Activities. Psychol. Sci..

[50] Mitchell T.R., Thompson L., Peterson E., Cronk R. (1997). Temporal adjustments in the evaluation of events: The “rosy view”. J. Exp. Soc. Psychol..

[51] Fritz C., Sonnentag S. (2005). Recovery, Health, and Job Performance: Effects of Weekend Experiences. J. Occup. Health Psychol..

[52] Mojza E.J., Lorenz C., Sonnentag S., Binnewies C. (2010). Daily recovery experiences: The role of volunteer work during leisure time. J. Occup. Health Psychol..

[53] Mojza E.J., Sonnentag S. (2010). Does volunteer work during leisure time buffer negative effects of job stressors? A diary study. Eur. J. Work Organ. Psychol..

[54] Bliese P.D., Lang J.W.B. (2016). Understanding relative and absolute change in discontinuous growth models Coding alternatives and implications for hypothesis testing. Organ. Res. Methods.

[55] Kanungo R.N. (1982). Measurement of job and work involvement. J. Appl. Psychol..

[56] Smit B.W., Barber L.K. (2016). Psychologically detaching despite high workloads: The role of attentional processes. J. Occup. Health Psychol..

[57] Derks D., Duin D., Tims M., Bakker A.B. (2015). Smartphone use and work–home interference: The moderating role of social norms and employee work engagement. J. Occup. Organ. Psychol..

[58] Van Dyne L., Cummings L.L., Parks J.M. (1995). Extra-role behaviors: In pursuit of construct and definitional clarity (A bridge over muddied waters). Res. Organ. Behav..

[59] Parker S.K., Griffin M.A. (2011). Understanding active psychological states: Embedding engagement in a wider nomological net and closer attention to performance. Eur. J. Work Organ. Psychol..

[60] Wrzesniewski A., Dutton J.E. (2001). Crafting a Job: Revisioning Employees as Active Crafters of Their Work. Acad. Manag. Rev..

